# Exploring educational needs on frailty outside geriatrics: a survey of European Union of Medical Specialists’ bodies

**DOI:** 10.1007/s41999-026-01469-z

**Published:** 2026-05-07

**Authors:** Román Romero Ortuño, Sara Solis López, M. Cristina Polidori, Tahir Masud, Michael Vassallo, Marianne van Iersel, Jūratė Macijauskienė, Eva Topinková, Maria Nuotio, Rozemarijn L. Van Bruchem-Visser, Maria Victoria Farré Mercadé, Mario Barbagallo

**Affiliations:** 1 Discipline of Medical Gerontology, School of Medicine, Trinity College Dublin, 6th Floor, Mercer’s Institute for Successful Ageing, St James’s Hospital, Dublin 8, Ireland; 2 Global Brain Health Institute, Trinity College Dublin, Dublin, Ireland; 3 Department of Medicine II, University Hospital of Cologne and PI Cluster of Excellence CECAD, University of Cologne, Cologne, Germany; 4 Nottingham University Hospitals NHS Trust, Nottingham, UK; 5 Bournemouth University, Dorset, UK; 6 Department of Geriatrics, Radboud University Medical Center, Nijmegen, The Netherlands; 7 Department of Geriatrics, Lithuanian University of Health Sciences, Kaunas, Lithuania

**Keywords:** Frailty, Aged, Health personnel, Education, Medical, Continuing, Surveys and questionnaires, Europe

## Abstract

**Purpose:**

Frailty in older adults is associated with increased vulnerability, poorer outcomes, and greater healthcare utilisation. To inform future inter-specialty educational initiatives, a survey across UEMS bodies was conducted.

**Methods:**

An online survey was disseminated between July and November 2025 via the UEMS Coordination team to all sections, divisions, multidisciplinary joint committees, and thematic federations. Eligible respondents were specialists not certified in Geriatric Medicine who provide care to older adults (≥ 65 years).

**Results:**

Of 416 respondents, 283 were non-geriatric specialists caring for older people. They encompassed 40 specialties (40% surgical, 33% medical, 15% emergency/critical care, 12% imaging/laboratory) across 39 countries (91% EU/EEA/UK). Respondents were predominantly late career (63%), university-hospital-based (73%), and 44% were female. Most (83%) reported no formal geriatric training. While the perceived importance of frailty was high (mean 8.0 out of 10), 56% believed frailty is generally or inevitably worsening over time. Although 68% were aware of at least one validated frailty tool, 36% never used such tools, most commonly due to lack of familiarity or time constraints. Confidence in managing frailty was low (mean 4.7/10), and only 29% felt frailty is adequately addressed within their clinical setting. Nearly all respondents agreed that non-geriatric specialists should have basic competencies in frailty (90%) and 86% supported including a frailty appendix in relevant European Training Requirements.

**Discussion:**

Non-geriatric specialists recognise the clinical importance of frailty, but report limited confidence and insufficient structured training. These findings underscore the need for accessible, standardised, inter-specialty frailty education to support high-quality care for older adults.

**Supplementary Information:**

The online version contains supplementary material available at 10.1007/s41999-026-01469-z.

## Key summary points

**Aim:**

To explore how non-geriatric specialists across UEMS bodies who care for older adults understand and approach frailty in order to guide future interspecialty educational initiatives.

**Findings:**

An online survey was conducted to capture their perspectives, practices and training needs regarding frailty.

**Message:**

Non-geriatric specialists recognise the importance of frailty but report limited confidence and training, indicating a clear need for accessible and standardised education.

**Supplementary Information:**

The online version contains supplementary material available at 10.1007/s41999-026-01469-z.

## Introduction

Frailty is a multidimensional geriatric syndrome that reflects a state of reduced physiological reserve and increased vulnerability to stressors in older adults [1, 2]. It is associated with higher risks of functional decline [3], hospitalisation [4], institutionalisation [5] and mortality [6], and contributes substantially to healthcare utilisation in ageing populations [7]. As the demographic profile of Europe continues to shift towards a greater proportion of older adults [8], recognition and management of frailty have become essential across the full spectrum of medical and surgical specialties [9, 10].

Although frailty is central to the discipline of Geriatric Medicine [11], older adults living with frailty are routinely encountered in many other specialties [12]. This creates a need for clinicians outside geriatrics to understand its clinical concept, to apply validated identification methods in assessment and to integrate frailty related considerations into clinical decision-making [13]. Over recent years, several validated frailty models and tools have been developed [14], and international guidelines increasingly recommend their use [15]. Despite this progress, the extent to which frailty knowledge and assessment practices are represented across non-geriatric specialties in Europe remains unclear [16]. Training in frailty for non-geriatric clinicians is variable, and formal educational pathways are not consistently incorporated into specialist curricula [13].

The mission of the European Union of Medical Specialists (UEMS) is to harmonise specialist training standards across Europe [17], and its Geriatric Medicine Section (UEMS-GMS) has identified frailty as a key cross-specialty competence in its European Training Requirements (ETRs) [18]. To inform future inter-specialty educational initiatives and potential updates to non-geriatric ETRs, it is necessary to understand how frailty is currently conceptualised and approached by specialists who routinely care for older adults but are not trained in Geriatric Medicine.

The aim of this study was to examine the perspectives, familiarity, current practices and educational needs related to frailty among non-geriatric specialists across UEMS bodies who provide care for older adults. This work seeks to provide an evidence base for strengthening frailty education across European specialist training and for supporting high-quality care for an ageing population.

## Methods

This study used a cross-sectional online survey that aimed to identify and understand the perspectives and educational needs regarding frailty in older adults among non-geriatric specialists affiliated with UEMS bodies.

### Setting

The UEMS is composed of 42 national associations that represent medical specialists in their respective countries. These National Member Associations fall into three categories: Full Members, which include member states of the European Union (EU), the European Economic Area (EEA) and the United Kingdom (UK); Associate Members, which include member states of the Council of Europe; and Observer Members, which include other countries. Within the UEMS structure, as of October 2024 [19], specialist activity is organised through 43 Specialist Sections (some of which have Divisions), 24 Multidisciplinary Joint Committees and 7 Thematic Federations, which collectively span the recognised medical and surgical specialties represented in the organisation. While UEMS Statutes foresee two delegates per country in each Section [20], in practice not all countries are represented in every body and when represented some countries appoint only one delegate. Yet, it is estimated that overall the UEMS represents more than 1.7 million specialist doctors across Europe and beyond. Its core objective is to improve patient care by developing and supporting excellence in specialist medical practice [19].

### Target participants

Eligible respondents were medical specialists within UEMS bodies who were not certified in Geriatric Medicine and who provided care for adults aged 65 years and older.

### Survey development

The survey was drafted by RRO, SSL and JM, and reviewed by members of the UEMS Geriatric Medicine Section. Screening questions at the start of the survey were used to exclude geriatricians and specialists who did not provide clinical care for older adults. The questionnaire included both closed and open-ended items [21]. It collected information on respondent characteristics including specialty, gender, years in practice, country of work and healthcare setting. Items assessed exposure to geriatrics training and familiarity with the concept of frailty. The survey then explored participants’ views on the epidemiology of frailty, its relevance to clinical practice and its perceived impact on patient and system outcomes. Additional sections addressed clinical recognition of frailty, signs and symptoms prompting frailty suspicion, and awareness of associations with other geriatric syndromes. Respondents were asked about routine identification of frailty, familiarity with validated frailty tools, frequency of tool use, perceived barriers and perceived added value of such tools. Further items examined experiences and challenges in managing frailty, confidence levels, management strategies used and views on whether frailty is adequately addressed in their clinical setting. Finally, the survey assessed educational needs, preferred formats for frailty training, priority topics for future education and support for incorporating basic frailty competencies into relevant ETRs.

### Survey dissemination and timeline

The survey, distributed as a Microsoft Forms link [21], was disseminated between July and November 2025 through the UEMS Coordination team. It was sent to all UEMS bodies following approvals from the UEMS-GMS, the UEMS Executive and the UEMS Advisory Board, as well as ethical approval, as documented in the Supplementary Table. To improve participation, several reminders were issued, as also detailed in the Supplementary Table. No incentives or compensation were offered for participation.

### Data handling and analysis

Anonymous survey data were exported from Microsoft Forms and stored on secure institutional servers at Trinity College Dublin, Ireland. Quantitative data were analysed using IBM SPSS Statistics for Windows, Version 29.0 (Armonk, NY: IBM Corp; 2022). Descriptive statistics were generated, including frequencies, percentages, means and standard deviations. All analyses reflected complete case data, and no weighting or imputation was applied.

Narrative responses to open-ended questions were analysed inductively. Initial coding and theme generation of fully anonymised data were supported by the use of ChatGPT 5.1 (OpenAI) [22] and the Free Word Cloud Generator online application (https://www.freewordcloudgenerator.com/). Review and verification of all outputs were performed manually by the research team to ensure that the identified themes accurately reflected the underlying data and that final theme labelling incorporated expert judgement and contextual interpretation.

### Representativity considerations

A priori, it was decided that analyses would first be presented for the full sample and then stratified by specialty group: medical, surgical, emergency or critical care, and imaging or laboratory. The response rate for each specialty was calculated using the reported size of the corresponding UEMS body, obtained either via personal communication with the designated contact for that body or through consultation of publicly available membership information on the body’s website. For the purposes of this study, and taking into account the email-based survey distribution [23], specialty-specific response rates were categorised as good (> 50%), acceptable (40–50%), suboptimal (20–39%), and very limited (< 20%). Sub-analyses were conducted only for specialties that met the study-defined threshold for an at least acceptable response rate. As the survey was disseminated centrally by the UEMS Coordination team and responses were anonymous, the exact number of recipients could not be determined and characteristics of non-responders were not available. Reporting of the survey was conducted in accordance with CHERRIES recommendations for online surveys [24].

### Ethical approval

Ethical approval was granted by the Trinity College Dublin School of Medicine Research Ethics Committee (REAMs No. 3953; 16 December 2024). The survey was anonymous and voluntary, and all data were stored securely at Trinity College Dublin in accordance with the approved protocol. The study adhered to the ethical principles of the 1964 Declaration of Helsinki and its later amendments.

## Results

### Respondents’ characteristics

**Fig. 1:**
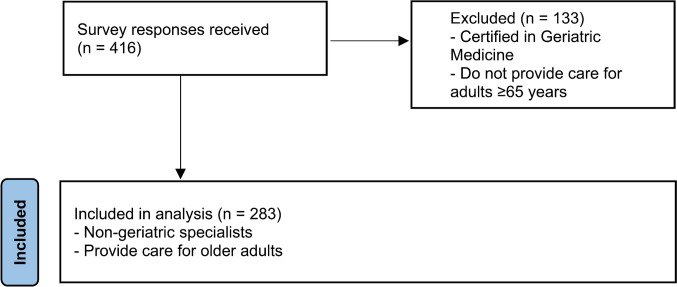
Flow diagram of participant selection

A total of 416 individuals responded to the survey. No instances of multiple entries were identified in the dataset. After applying eligibility screening, 133 respondents were excluded because they were certified in Geriatric Medicine or did not provide clinical care for adults aged ≥ 65 years (Fig. 1). The 283 eligible participants required a mean (SD) of 20.8 (20.5) minutes to complete the survey. Respondents were based in 39 countries, with 83.0% located in EU or EEA countries and a further 8.2% in the UK. Overall, 43.8% of respondents were female, and most were senior clinicians, with 62.5% having ≥ 21 years of experience and an additional 27.2% having 11–20 years of experience. The majority (73.1%) worked in a university hospital setting, 82.7% reported no previous formal education or training in geriatrics or the care of older adults, and 89.4% reported no previous formal training specifically in frailty identification and management.

### Breakdown of specialties

Respondents encompassed 40 specialties, grouped as surgical (*n* = 113, 39.9%), medical (*n* = 93, 32.9%), emergency or critical care (*n* = 42, 14.8%), and imaging or laboratory disciplines (*n* = 35, 12.4%). Table 1 presents the breakdown of individual specialties and their response rate relative to the reported size of each corresponding UEMS body. Two specialties met the study’s criterion for an acceptable response rate (40–50%), namely General Surgery (54.9%) and Emergency Medicine (43.6%). Several specialties demonstrated suboptimal response rates (20–39%), namely Breast Surgery (34.5%), Urology (33.3%), Nephrology (26.7%), Angiology/Vascular Medicine (25.0%), Endocrinology (24.2%), Infectious Diseases (23.6%), Otorhinolaryngology (21.9%), Anaesthesiology (21.4%), Radiology (20.6%), and Endocrine Surgery (20.0%). All other specialties showed very limited response rates (< 20%). By specialty groups, emergency and critical care demonstrated the highest overall response rate (24.5%), followed by surgical specialties (17.1%) and imaging or laboratory disciplines (14.3%), while medical specialties showed the lowest overall representativity (10.0%).

**Table 1:** Response rates of individual UEMS specialties by specialty group. Body size represents the estimated number of delegates/members in each body

		Body size	Number of replies	Response rate (%)
Surgical	General surgery	51	28	54.9
	Breast surgery	29	10	34.5
	Urology	54	18	33.3
	Otorhinolaryngology	64	14	21.9
	Endocrine surgery	5	1	20.0
	Vascular surgery	63	12	19.0
	Plastic, reconstructive and aesthetic surgery	55	6	10.9
	Oro-maxillo-facial surgery	40	4	10.0
	Thoracic surgery	62	6	9.7
	Ophthalmology	66	4	6.1
	Transplant surgery	52	3	5.8
	Orthopaedics and traumatology	55	3	5.5
	Obstetrics and gynaecology	50	2	4.0
	Neurosurgery	52	2	3.8
Medical	Nephrology	30	8	26.7
	Angiology/vascular medicine	32	8	25.0
	Endocrinology	62	15	24.2
	Infectious diseases	55	13	23.6
	Internal medicine	54	10	18.5
	Physical and rehabilitation medicine	95	17	17.9
	Cardiology	41	5	12.2
	Public health	37	2	5.4
	Dermatology and venerology	57	3	5.3
	Medical oncology	20	1	5.0
	Wound healing	20	1	5.0
	Clinical genetics	51	2	3.9
	Allergology	64	2	3.1
	Medical clinical pharmacology	32	1	3.1
	Phlebology	33	1	3.0
	Rheumatology	48	1	2.1
	Respiratory medicine	50	1	2.0
	Medical microbiology	54	1	1.9
	Gastroenterology and hepatology	69	1	1.4
Emergency/critical care	Emergency medicine	55	24	43.6
	Anaesthesiology	70	15	21.4
	Intensive care medicine	36	3	8.3
Imaging/laboratory	Radiology	63	13	20.6
	Nuclear medicine	81	15	18.5
	Interventional radiology	36	5	13.9
	Laboratory medicine	47	2	4.3

### Understanding of frailty

**Fig. 2:**
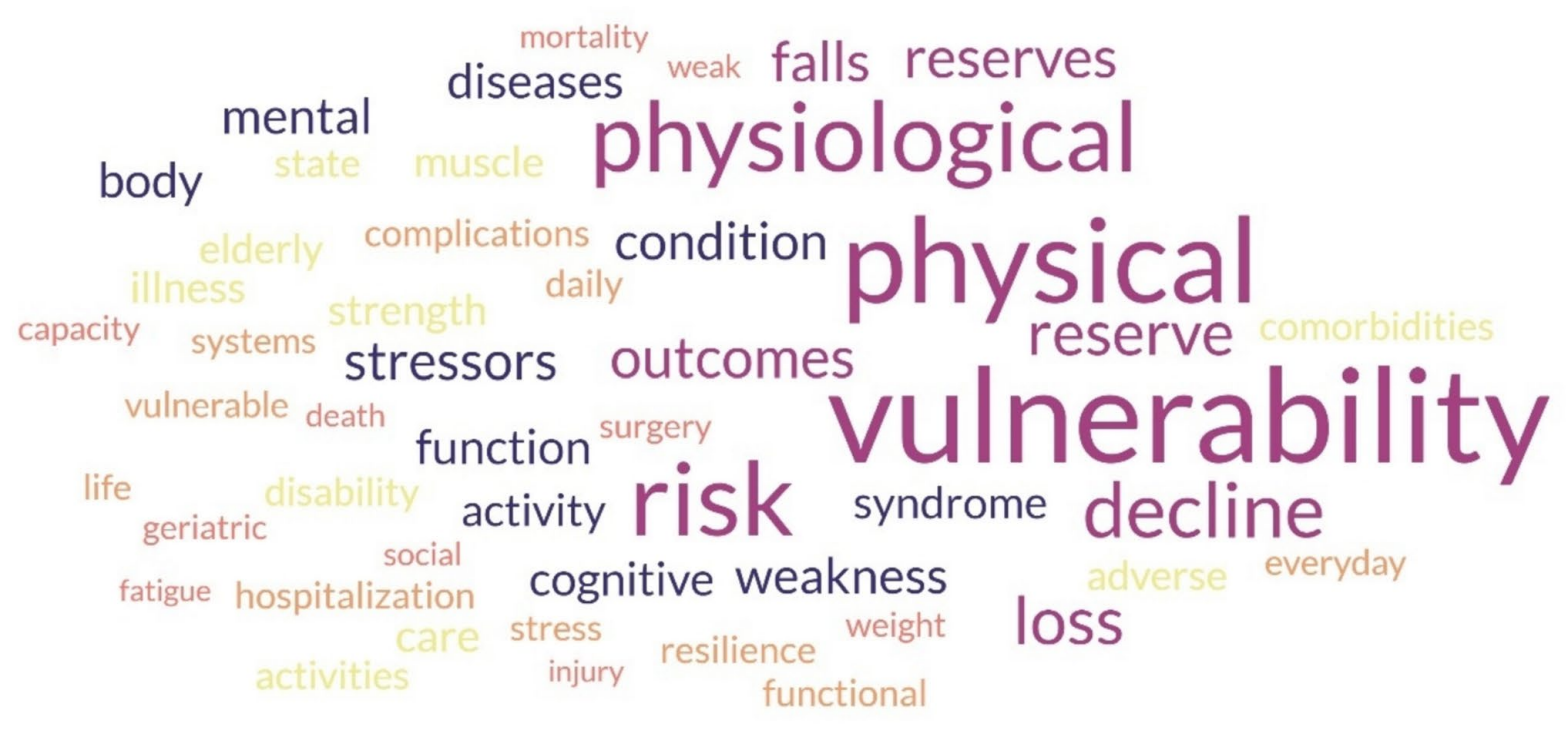
Word cloud of respondents’ free-text definitions of frailty. “*In your own words, please provide a brief definition of frailty in older adults based on your current understanding*.” Larger words reflect higher frequency

Eligible participants reported a high level of self-rated familiarity with the concept of frailty in older adults. On a scale from 0 (not at all familiar) to 10 (extremely familiar), the mean (SD) score was 7.3 (2.0). Respondents’ open-ended definitions of frailty (*n* = 272) emphasised themes of vulnerability, reduced physical and physiological reserves, and increased risk of adverse outcomes, as illustrated in the word cloud depicting the 50 most frequently cited terms (Fig. 2).

In response to the question “*In your experience, which patients are most likely to be considered frail?*”, all respondents (100%) selected patients with multiple chronic conditions. Physical disabilities (82%), cognitive disabilities (79%), and terminal diagnoses (74%) were also frequently endorsed, while fewer than 5% identified an “other” category. The latter included advanced age, malnutrition, polypharmacy, chronic kidney disease or dialysis, dementia, serious recent illness or injury, mental illness, depression, social isolation, and broader social determinants such as poor socioeconomic conditions and lack of family support.

When asked, ‘*What are the key signs or symptoms that make you suspect frailty in a patient?*’, respondents most commonly selected muscle weakness (98.6%), slow walking speed (97.2%), fatigue (96.8%), low physical activity (95.1%), unintentional weight loss (94.7%), sarcopenia (92.9%), and cognitive problems (81.3%), while obesity was less frequently endorsed (38.9%). Only 30 respondents (10.6%) selected an ‘other’ option; these free-text responses highlighted additional indicators such as difficulties with activities of daily living, recurrent falls, functional decline, malnutrition, delayed healing, polypharmacy, repeated hospitalisations, depression and apathy, and social isolation.

Respondents were also asked about awareness of the associations of frailty with other geriatric syndromes. Awareness (i.e. selected aware or very aware) was greatest for falls (89.4%), multimorbidity (88.3%), and dementia (82.7%), followed by malnutrition (80.6%), polypharmacy (78.4%), mobility impairment (78.1%), and loss of skin integrity (77.0%). Moderate levels of awareness were reported for delirium (65.4%) and loneliness (65.4%), as well as mood disorders such as depression or anxiety (62.2%). Lower awareness was noted for sensory deficits (57.6%) and was lowest for incontinence or constipation (56.9%).

### Perceived epidemiology of frailty

Only 26.9% of respondents agreed or strongly agreed that frailty is a universal condition in adults over 65 years, while the majority selected disagree or strongly disagree (57.6%), with the remainder choosing a neutral option. The vast majority (93.3%) agreed or strongly agreed that frailty becomes more common with increasing age but is not present in all older adults. Views were more divided regarding community-dwelling older people: 46.6% agreed or strongly agreed that most adults over 65 living in the community are not frail. In contrast, there was broad consensus about institutional settings, with 86.9% agreeing or strongly agreeing that the majority of nursing home residents live with frailty.

Respondents most commonly perceived frailty as a condition that generally worsens over time, with periods of stability (45.9%). A further 17.7% viewed frailty as fluctuating, with the potential to improve or deteriorate depending on circumstances. Just over one quarter (25.8%) believed frailty can improve with appropriate interventions. In contrast, 9.9% felt frailty inevitably worsens, and only 0.7% selected “other.” Overall, more than half (55.8%) believed that frailty in older adults generally or inevitably worsens over time.

### Perceived lived experienced of frailty

Most respondents felt that older adults living with frailty often perceive themselves as more dependent on others (87.6%), less confident or capable (78.4%), frustrated or burdened by their limitations (69.9%), and fearful of further decline (67.4%). A small proportion perceived them as resilient despite challenges (18.4%) or content and accepting of their condition (12.4%), while 2.8% selected an “other” description. The free-text “other” responses described a wide range of emotional experiences, including insecurity, anxiety, loneliness, depressive feelings, desperation, or acceptance that varied greatly depending on personal circumstances and available support.

### Perceived importance and impact of frailty on outcomes

Respondents rated frailty as highly important in their clinical practice, with a mean (SD) score of 8.0 (1.7) on a 0–10 scale. The majority of respondents (77.7%) agreed with the statement that frailty should be a formal *diagnosis* in clinical practice, while 22.3% did not support its formal diagnostic status. Respondents who supported frailty as a formal diagnosis consistently emphasised its substantial impact on prognosis, risk stratification, clinical decision-making, care planning, communication, and resource allocation, noting that formal recognition would improve early identification, guide personalised interventions, enhance patient and family understanding, and promote more coordinated, preventative, and evidence-based care. Those who opposed frailty being a formal diagnosis largely argued that it is not a discrete disease but a multifactorial geriatric syndrome or age-related condition arising from diverse underlying causes, cautioning that formalising it could risk oversimplification, over-labelling, or ageism, obscure treatable contributors, and medicalise normal ageing, noting instead that frailty should be assessed, recognised, and managed without being classified as a standalone diagnosis.

Most participants believed that frailty has a substantial influence on a wide range of patient outcomes: major or significant impact was attributed to the ability to withstand an acute illness (88.7%), mortality (91.8%), treatment complications (91.2%), care planning (84.5%), and admission to a nursing home (83.4%). Perceived impact on care satisfaction was more moderate, with 51.2% reporting a major or significant effect. Frailty was likewise viewed as highly consequential for system-level outcomes, with major or significant impact reported for unplanned hospital admissions (89.8%), hospital length of stay (91.2%), unplanned readmissions (90.1%), and cost of care (88.7%).

### Frailty identification practices

Overall across the full sample (*n* = 283), self-reported confidence in using frailty identification tools was modest (mean 4.3 out of 10, SD 3.2). Most respondents (71.4%) reported routinely identifying frailty in their clinical practice, and 67.5% were aware of at least one validated frailty identification tool. Across the full sample, the Clinical Frailty Scale (CFS) was the most widely recognised tool, selected by 47.0% of respondents. The Frailty Index was the next most frequently identified (38.2%), followed by the FRAIL Scale (18.4%), the Physical (Fried’s) Frailty Phenotype (15.2%), and the Edmonton Frail Scale (EFS) (15.2%). The PRISMA-7 questionnaire was less commonly endorsed (10.2%). Approximately 6% of respondents reported additional tools in free-text responses, each mentioned by fewer than 1% individually. These included a diverse range of geriatric, functional, and comorbidity assessments, such as the Charlson Comorbidity Index, ECOG, G8 screening tool, Groningen Frailty Scale, hand-grip strength or gait speed, the modified Rankin Scale, and the VES-13.

Across respondents, the most widely perceived benefit of frailty identification tools was their ability to help clinicians identify at-risk patients early, selected by 67.0% of participants. A similarly large proportion reported that tools support decision-making about interventions (62.1%) and aid in tailoring treatment and care plans (56.7%). Nearly half indicated that frailty tools improve communication with patients and families about health risks (46.1%) and enhance interdisciplinary care coordination (48.9%).

More than one-third (36.0%) reported never using frailty identification tools in practice. The most frequent barriers to using tools were lack of familiarity, insufficient time, and that tools were impractical or unnecessary, especially in imaging or laboratory specialities.

### Frailty management approaches

Overall across the full sample (*n* = 283), self-reported confidence in managing frailty was modest (mean 4.7 out of 10, SD 2.7). Clinicians reported using a wide range of strategies to manage frailty. The most frequently selected approach was a multidisciplinary team (MDT) approach, endorsed by 69.3% of respondents. Nutritional support was also commonly used (53.7%), followed by medication review and optimisation (48.4%) and referral to specialist geriatric medicine services (47.3%). Fall-prevention strategies were selected by 43.8%, and 39.6% reported using structured exercise programmes such as strength training. Cognitive assessments and interventions were implemented by 36.4%.

Only 29% of respondents agreed that frailty was adequately addressed within their clinical setting. Respondents reported a wide range of challenges in managing frailty in older adults. The most frequently cited barrier was limited time for comprehensive assessments, reported by 51.2% of participants. This was closely followed by the lack of standardised protocols (49.5%) and difficulty coordinating care with other providers (48.4%). A substantial proportion also identified the limited availability of geriatricians (47.0%) and uncertainty about how best to treat frailty (40.6%) as major challenges. Additionally, managing multiple co-existing conditions alongside frailty was reported by 39.9% of respondents. Less commonly, 13.1% indicated that frailty is not viewed as a core component of clinical practice within their specialty.

Furthermore, reported barriers to managing frailty were highly prevalent among respondents. The most frequently reported challenge was lack of resources—such as limited time or staffing—cited by 67.8% of participants. Lack of training or knowledge was similarly common (63.6%), followed by reduced availability of geriatricians (51.2%) and broader systemic or structural challenges, including organisational or policy constraints (43.5%). Resistance from patients or families was selected by a much smaller proportion (13.4%).

When asked what additional resources would help improve frailty management in their clinical practice, respondents most frequently highlighted the need for guidelines and protocols on frailty management, selected by 71.0% of participants. Over half (54.1%) reported that improved access to frailty identification tools would support their practice, while 47.0% expressed a need for better collaboration with geriatricians, emphasising multidisciplinary integration. Additionally, 44.2% indicated that having more time available for frailty assessments would meaningfully enhance their ability to deliver appropriate care.

### Educational needs and training preferences

There was overwhelming agreement among respondents that non-geriatricians should possess basic competences in identifying and managing frailty. Almost nine in ten (89.8%) endorsed this view, including 57.2% who strongly agreed and 32.5% who agreed. Only 10.2% expressed neutrality or disagreement.

Overall, 79.9% indicated they would like to receive education or training on frailty, while 20.1% reported no interest. Online courses were the most widely favoured option, selected by 62.9% of participants, followed by webinars (50.5%), indicating a strong preference for virtual, scalable formats. Around one-third (32.9%) favoured in-person workshops, while 25.4% preferred conferences or symposia. Self-paced modules were endorsed by 22.6%.

Respondents indicated clear priorities regarding the content of frailty education. The most frequently selected topics were multidisciplinary approaches to managing frailty (64.7%), early recognition of frailty (62.9%), and frailty identification tools (61.1%). Preventive measures and interventions (37.1%) and comorbidity management (33.9%) were also commonly chosen. Fewer participants selected palliative care and frailty (20.8%) or cognitive and psychological aspects of frailty (15.5%).

Most respondents (85.9%) supported adding an appendix to all relevant European Training Requirements to define basic competencies in frailty identification and management. Only 14.1% were opposed.

Table 2 summarises the survey results across key metrics, presented overall, by specialty group, and with separate breakdowns for general surgery and emergency medicine, the two specialties with the highest response rates. Although familiarity with frailty and recognition of its clinical importance were generally high, confidence in both identifying and managing frailty remained low—particularly among imaging/laboratory specialists—while emergency medicine respondents reported the highest confidence levels. Use of frailty identification tools varied widely, with substantial non-use in several groups, and formal training in geriatrics or frailty was uncommon across all specialties. Despite these gaps, there was near-universal agreement that non-geriatricians should have basic frailty competencies, strong interest in receiving further training, and broad support for incorporating a frailty appendix into European Training Requirements.

**Table 2:** Key survey findings across specialty groups

		Overall (*n* = 283)	Medical (*n* = 93)	Surgical (*n* = 113)	Emergency/critical care (*n* = 42)	Imaging or laboratory (*n* = 35)	Emergency medicine (*n* = 24)	General surgery (*n* = 28)
Respondents’ characteristics	Mean time to complete the survey, minutes (SD)	20.8 (20.5)	22.1 (19.7)	19.0 (16.7)	18.9 (14.1)	25.4 (35.1)	19.5 (15.9)	17.7 (12.2)
	Based in EU/EEA/UK (%)	91.2	88.2	92.9	85.7	100.0	91.7	82.1
	Female (%)	43.8	57.0	31.0	50.0	42.9	33.3	10.7
	≥ 21 years of experience (%)	62.5	67.7	60.2	61.9	57.1	45.8	50.0
	Worked in university hospital (%)	73.1	74.2	70.8	71.4	80.0	75.0	60.7
	No previous formal education/training in geriatrics/care of older adults (%)	82.7	79.6	88.5	71.4	85.7	66.7	82.1
	No previous formal education/training in frailty identification/management (%)	89.4	90.3	92.9	73.8	94.3	62.5	89.3
Understanding of frailty	Mean familiarity with the concept of frailty (SD)	7.3 (2.0)	7.3 (2.0)	7.1 (2.1)	8.2 (1.4)	6.5 (2.0)	8.5 (1.4)	7.7 (1.9)
Clinical importance of frailty	Mean importance of frailty in clinical practice (SD)	8.0 (1.7)	7.9 (1.6)	8.2 (1.4)	8.6 (1.4)	7.0 (2.6)	8.9 (1.2)	8.3 (1.4)
Frailty identification	Mean confidence in using frailty identification tools (SD)	4.3 (3.2)	4.5 (3.2)	4.2 (3.2)	6.5 (2.8)	2.1 (2.6)	7.6 (1.9)	5.6 (2.7)
	Never uses frailty identification tools in practice (%)	36.0	38.7	29.2	9.5	82.9	0.0	7.1
Frailty management	Mean confidence in managing frailty (SD)	4.7 (2.7)	5.1 (2.6)	4.2 (2.6)	5.8 (2.3)	3.5 (2.7)	6.2 (1.9)	5.2 (2.2)
	Frailty is adequately addressed in the clinical setting (%)	29.0	34.4	26.5	21.4	31.4	16.7	32.1
Frailty education	Non-geriatricians should have basic frailty competencies (%)	89.8	91.4	88.5	100.0	77.1	100.0	78.6
	Interested in receiving education/training on frailty (%)	79.9	78.5	80.5	85.7	74.3	87.5	78.6
Appendix to ETRs	Supports adding a frailty appendix to all relevant ETRs (%)	85.9	81.7	86.7	95.2	82.9	100.0	85.7

## Discussion

This cross-specialty survey provides the first detailed examination of how frailty in older adults is perceived and approached outside Geriatric Medicine across UEMS bodies. The results reveal that although frailty is recognised as conceptually familiar and clinically important, its practical identification and perceived management remain uneven and limited. However, the findings also indicate a high degree of openness to further education and cross-specialty collaboration, suggesting strong potential for strengthening frailty-informed practice across non-geriatric specialties that provide care for older adults.

Of the 416 individuals who responded to the survey, 283 (68.0%) met the predefined eligibility criteria of being non-geriatric specialists who provide clinical care for adults aged ≥ 65 years. The remainder were primarily geriatricians who, although outside the intended target population, engaged with the survey, a pattern likely reflecting the practical constraints of distributing the survey through the Coordination team to all UEMS bodies simultaneously rather than pre-filtering recipients.

Most eligible respondents were late-career clinicians. This distribution likely reflects the structure of UEMS bodies, where delegates typically represent experienced specialists within their national societies, resulting in limited representation of early-career physicians. However, this also makes the finding that 82.7% had received no previous formal training in geriatrics and 89.4% no formal training in frailty particularly notable. These proportions suggest that limited exposure to formal geriatric education outside geriatrics is a still a longstanding issue [25] that extends even to senior specialists, and may be still more heterogeneous in samples that include a higher proportion of junior specialists.

Despite very limited *formal* training, respondents demonstrated relatively high familiarity with the concept of frailty. This was evident in both quantitative ratings and the quality of free-text definitions. Many descriptions aligned with the established frailty definition, emphasising reduced physiological reserve, vulnerability to stressors, and increased risk of adverse outcomes [1, 2]. This conceptual alignment may reflect substantial informal learning acquired through clinical practice, interdisciplinary collaboration, and the much more visible academic discourse on frailty in the past 20 years [26–28]. Future studies could further explore the specific pathways through which non-geriatric specialists acquire knowledge about frailty outside formal training programmes, including experiential learning, peer collaboration and engagement with the academic literature.

Respondents accurately recognised that frailty becomes more common with advancing age and is not universal in people aged 65 years and older. However, their views on its prevalence in the community were more pessimistic than the evidence suggests: while only 46.6% agreed or strongly agreed that most adults over 65 living in the community are not frail, robust epidemiological data indicate that no more than one quarter of this population live with frailty [29, 30]. This suggests a degree of overestimation influenced by hospital-based clinical experience, where frailty prevalence is naturally higher [31].

Additionally, more than half of respondents believed that frailty generally or inevitably worsens over time. Although this perception is not uncommon among clinicians [32] and mirrors public understanding [33, 34], it does not fully reflect the emerging literature on frailty transitions, which shows that improvement or stability is not only possible, but more frequent than commonly assumed [35–40]. These findings highlight an opportunity for education focussing on the dynamic and potentially reversible elements of frailty and on the value of timely, targeted interventions [41].

Respondents frequently associated frailty with disability, dependence, loss of confidence, frustration and fear of further decline. Yet, evidence suggests that the lived experience of people identified as frail in the community is not uniformly negative, as many report good or very good health, relatively high quality of life and sustained engagement in social activities [42]. These perceptions among clinicians correspond to a predominantly disability-oriented model of frailty, which may reflect the types of cases encountered in secondary and tertiary care settings [43]. However, this model risks overshadowing earlier, pre-disability stages of frailty (e.g. physical frailty) [44], where interventions may be particularly effective [39]. Educational strategies should therefore address recognised differences (even if overlaps exist) between frailty and disability [45, 46] and promote awareness of the more positive and heterogeneous aspects of the lived experience [47].

A large majority of respondents supported the idea of frailty as a formal diagnosis. While this may reflect recognition of frailty as clinically meaningful in their practice, it may also reflect ambiguity within the literature, where frailty has often been described or implied as a diagnosis [48–50]. Yet, as a minority of respondents correctly recognised, frailty is a multidimensional clinical state rather than a discrete diagnosis, and appropriate management depends on identifying and diagnosing the underlying conditions that give rise to it [46, 51]. Educational efforts should therefore emphasise frailty as a clinical state that prompts full evaluation, informs prognosis and guides care planning, rather than a diagnosis in itself.

Respondents reported low confidence in using frailty identification tools despite broad awareness of the concept. From a Geriatric Medicine perspective, this is understandable, as even within the specialty there is no single universally adopted frailty tool, despite early academic exploration of the issue [52]. It is now widely recognised that different frailty instruments identify different patient profiles and are variably suited to specific settings and purposes [53, 54]. Additionally, the survey suggests that familiarity with specific frailty tools may vary across specialties, with the Clinical Frailty Scale more commonly recognised in acute care, the Edmonton Frail Scale in preoperative assessment and the G8 in oncogeriatric practice. These specialty-specific tendencies may reflect differing academic influences and established local practices, but they could also contribute to limited awareness of alternative tools that might offer greater added value in certain clinical contexts. Providing structured guidance on the general appraisal and selection of frailty tools [55, 56] could therefore support clinicians in making informed, context-appropriate decisions.

Confidence in managing frailty was low, and many respondents reported that frailty was not adequately addressed within their clinical settings. This is consistent with perceptions of limited time, interdisciplinary support, and access to specialist geriatric inputs. These challenges reflect broader structural issues within health systems, where the availability of geriatric services does not fully match the growing needs of an ageing population. As specialists in Geriatric Medicine cannot realistically provide direct care for all older adults, there is a need to prioritise geriatrics within the education of all clinicians [57], and to support the dissemination of effective practices and core skills across disciplines [58]. Enhancing liaison geriatric models and improving access to multidisciplinary teams may help clinicians integrate frailty-informed approaches more consistently into routine care [59, 60].

Despite the challenges identified, there was strong enthusiasm for frailty education. Most respondents expressed interest in training, particularly via flexible online platforms, which aligns with existing educational offerings in this area [61–65]. Evidence indicates that frailty education programmes, although still limited in number, are generally well received and effective in improving knowledge and self-perceived competence, particularly when delivered through flexible, clinically tailored and multidisciplinary formats [13]. The 2014 European Undergraduate Curriculum in Geriatric Medicine recommended frailty training for medical students and is currently being updated, which should help ensure future medical graduates have better knowledge of frailty [66]. The vast majority of respondents supported adding frailty competencies to the ETRs of relevant specialties, providing a clear mandate for coordinated action at the UEMS level. The UEMS-GMS is well positioned to lead this effort by utilising the information gathered in this survey to co-develop a cross-specialty appendix that defines foundational competencies and supports specialty-adapted implementation.

A strength of this survey is its focus on UEMS bodies, which represent a well-defined, experienced and pan-European cohort of specialists who officially represent their countries. The survey achieved broad disciplinary reach and generated rich quantitative and qualitative data, complementing similar evidence from single-country studies [67]. Another notable strength is that the reporting of the survey results adheres to recognised guidance on survey methodology, including recommended practices for transparent and rigorous reporting [68].

Response rates varied widely across UEMS specialties, and this variability has implications for representativity. Although no universal consensus exists on minimum acceptable survey response rates [69], only General Surgery and Emergency Medicine met the study’s predefined threshold for an acceptable response rate, and none approached the more stringent “60% rule” described in the literature as an ideal benchmark [70, 71]. These differences partly reflect variation in the size, activity and engagement levels of individual UEMS bodies. As with all voluntary surveys, nonresponse bias remains likely, particularly in specialties with limited replies. The survey was relatively long, requiring a mean of 21 min to complete, which may have contributed to lower participation in some specialties, despite multiple reminders and invaluable support from the central UEMS Coordination team. However, this level of detail was necessary to obtain sufficiently nuanced insight into attitudes, understanding and practices across a diverse specialist workforce. The reliance on self-reported data introduces the potential for recall and social desirability biases. In addition, several questions used prompted ‘select all that apply’ response options, which may have increased the likelihood of endorsing certain associations compared with unprompted formats. As a cross-sectional snapshot, the design cannot assess changes in knowledge or practice over time. Although respondents represented 39 countries, the number of participants per country was generally small, which limited the ability to explore potential differences related to national training structures or healthcare systems. The relatively small number of respondents with prior formal training in geriatrics or frailty also limited our ability to examine the possible association between such training and knowledge, awareness, or confidence. Finally, Family Medicine was not represented in this survey because it is not organised within UEMS specialist Sections but through the European Union of General Practitioners, (UEMO). As a result, perspectives from this important discipline were not captured in the present study.

As Nicholas Coni reflected in 1996, “*Geriatrics is too important to be left to geriatricians*” [72]. Although offered almost 3 decades ago, this sentiment remains highly relevant: equipping all clinicians with core frailty competencies is critical to meeting the needs of ageing populations. This UEMS survey provides a strong foundation on which to advance harmonised frailty education across non-geriatric specialties in Europe.

## Supplementary Information

Below is the link to the electronic supplementary material.

Supplementary file1 (DOCX 22 KB)

## Data Availability

The data underlying this study were derived from an anonymous online survey conducted among representatives of UEMS bodies. Due to the nature of the data and the potential risk of indirect identification of respondents, the dataset is not publicly available, in accordance with the approval granted by the Trinity College Dublin School of Medicine Research Ethics Committee.
