# Supplementary material for: Exploring educational needs on frailty outside geriatrics: a survey of European Union of Medical Specialists’ bodies
Source: Eur Geriatr Med. 2026 May 7;17(3):1319–32. doi: 10.1007/s41999-026-01469-z (PMC13309420; doi:10.1007/s41999-026-01469-z)
Supplement: Supplementary file 1 — Supplementary file1 (DOCX 22 KB) [file 41999_2026_1469_MOESM1_ESM.docx]

**Supplementary Table.** Survey dissemination and key milestones

| Date | Event / Milestone |
| --- | --- |
| 26 Jun 2024 | Proposal for survey on frailty submitted to the UEMS Executive |
| 7 Aug 2024 | UEMS Executive formally supports the proposal |
| 21 Sep 2024 | UEMS-GMS meeting, Valencia, proposal endorsed |
| 18 Oct 2024 | Initiative introduced at the UEMS Advisory Board Meeting, Brussels |
| 16 Dec 2024 | Ethical approval granted by Trinity College Dublin School of Medicine REC (REAMs No. 3953) |
| 9 Apr 2025 | Draft survey presented at UEMS-GMS and approved for dissemination |
| 28 Jun 2025 | Survey link and circulation request issued to UEMS Executive |
| 10 Jul 2025 | Survey officially released to UEMS bodies by the UEMS Coordination team |
| 11 Jul 2025 | Contact list of UEMS bodies obtained from Coordination |
| 15 Jul 2025 | Named contacts emailed to gather support and confirm size of each UEMS body |
| 21 Aug 2025 | First central reminder issued by UEMS Coordination |
| 30 Aug 2025 | Reminder sent to named contacts |
| 20 Sep 2025 | Second central reminder issued by UEMS Coordination |
| 18 Oct 2025 | Reminder provided at the UEMS Autumn Council Meeting, Tbilisi |
| 6 Nov 2025 | Survey closed |

UEMS: European Union of Medical Specialists; GMS: Geriatric Medicine Section; REC: Research Ethics Committee.
